# Energy-Aware Duty Cycle Management for Solar-Powered IoT Devices

**DOI:** 10.3390/s25144500

**Published:** 2025-07-19

**Authors:** Michael Gerndt, Mustafa Ispir, Isaac Nunez, Shajulin Benedict

**Affiliations:** 1 Chair of Computer Architecture and Parallel Systems, School of Computation, Information and Technology, Technical University of Munich, 80333 Munich, Germany; orkun.ispir@tum.de (M.I.); isaac.nunez@tum.de (I.N.); 2 Department of Computer Science and Engineering, Indian Institute of Information Technology Kottayam, Valavoor P.O., Kottayam District, Kottayam 686635, Kerala, India; shajulin@iiitkottayam.ac.in

**Keywords:** computing continuum, duty cycle, energy harvesting, internet of things, serverless

## Abstract

IoT devices with sensors and actuators are frequently deployed in environments without access to the power grid. These devices are battery powered and might make use of energy harvesting if battery lifetime is too limited. This article focuses on automatically adapting the duty cycle frequency to the predicted available solar energy so that a continuous operation of IoT applications is guaranteed. The implementation is based on a low-cost solar control board that is integrated with the Serverless IoT Framework (SIF), which provides an event-based programming paradigm for microcontroller-based IoT devices. The paper presents a case study where the IoT device sleep time is pro-actively adapted to a predicted sequence of cloudy days to guarantee continuous operation.

## 1. Introduction

With a steep rise in battery-powered IoT sensors based on energy-efficient microcontrollers, the design of appropriate energy-conscious software for sensors is of growing importance. In many scenarios, a small form factor limits battery size and solar panel size for energy harvesting. For these sensors, it is important to adapt energy usage to the availability of solar energy under the constraints of the application requirements. Example scenarios are remote environmental monitoring and smart agriculture where sensors are deployed without access to the power grid and continuous operation is important.

IoT devices operate in a duty cycle, which executes the applications, and a sleep cycle in between duty cycles. The frequency of the duty cycle is dependent on the application. It can range from every few milliseconds for control tasks to several minutes or hours for sensors collecting environmental data.

This paper focuses on IoT-enabled sensors that use energy harvesting based on solar panels for attaining continuous operation. The duty cycle frequency of such devices is automatically controlled by setting the sleep time in the interval of the preferred minimum sleep time and the tolerated maximum sleep time. During the sleep time, microcontrollers are in deep sleep mode, which shuts down large fractions of the chip except the base clock and the logic required for interrupt handling. Accordingly, RAM contents get lost, and the microcontroller has to reboot the application whenever it wakes up. Deep sleep requires much less energy and significantly affects the *Remaining State of Charge (RSOC)* of the battery.

Designing IoT-enabled applications that self-sustain on power sources by combining energy harvesting techniques, a battery, and management software comes with a few notable challenges as listed below:

1. Inefficient Energy Harvesting Solutions: The energy harvesting solutions from solar, piezoelectric, photovoltaic, thermal, and so forth have to provide the maximum power transfer efficiency. In several existing methods, there are hardware mismatches that lead to failures.
2. Non-Availability of Integrated Software—Embedded IoT solutions use tiny devices with severe limitation in computational power and memory. Therefore, also the energy management code has to respect these limitations and integrate well with the application code to reduce additional overheads.
3. Increased Application Complexity—Developers tend to skip the handling of energy consumption due to the increased complexity of the application.

In this article, we propose energy-aware duty cycle management for IoT devices to attain continuous operation. It combines online sunshine predictions from a weather service with device-level energy measurements, a battery and a solar panel model to dynamically adapt the duty cycle frequency so that continuous operation for a given number of days and a certain *Remaining State of Charge (RSOC)* of the internal battery after this period can be guaranteed.

Our research questions are as follows:

1. Which information and algorithms are required to dynamically adapt the duty cycle frequency of solar-powered IoT sensors to mitigate fluctuations in the availability of solar energy?
2. How can the required information be collected with off-the-shelf, low-cost hardware?
3. How can duty cycle management be integrated into IoT applications without creating additional challenges in application development?

The rest of the article is structured as follows: Section 2 presents the existing research works carried out in the field of embedded IoT and energy optimization; Section 3 describes the architecture of the proposed energy management system; it presents the PCB developed for gathering energy data in Section 3.3; Section 4 introduces the models for our energy-aware duty cycle management and presents the adaptation algorithm; Section 5 reveals the experimental setup and the integration of the energy-aware duty cycle management into applications. The experiments demonstrate the effectiveness of the solution by proactively adapting the duty cycle to predicted weather changes such that enough energy is stored in the battery for the coming days without sunshine. Finally, Section 6 highlights a few research thoughts about the proposed work and future directions.

## 2. Related Works

IoT-enabled applications and associated hardware devices increased drastically in the past in various domains such as transport [1], industry, agriculture [2], education, healthcare, and so forth. Flexible, battery-powered tiny devices enabling multi-sensor connectivity and data privacy for a long duration require energy-efficient [3] solutions.

Traditionally, energy efficiency in embedded IoT devices has multiple dimensions: (a) varying voltage and clock frequencies; (b) switching ON/OFF device components; (c) monitoring and tuning the energy consumption of IoT devices [4]; (d) adopting energy harvesting techniques; and (e) developing low-power hardware designs. For instance, the authors of [5] studied the impact of varying sleep modes on IoT-based microcontrollers; similarly, the authors of [6] investigated the energy consumption of devices and the lifetime capacity of batteries. In [7], the authors proposed a three-transistor logic-based energy management system to minimize the energy consumption of embedded IoT devices.

Among energy harvesting methods such as thermal, vibration, and piezoelectric harvesting methods, solar-based energy harvesting has been applied in sensor-enabled applications due to high yield and predictability [8,9,10].

As microcontrollers have become more powerful, machine learning models are nowadays deployed on tiny devices as well [11,12]. Several approaches use machine learning for controlling energy consumption with respect to the availability of renewable energy. Notably, the authors of [13] developed a data transmission control for low-power IoT devices. It is based on fuzzy rules that are adapted in an evolutionary fashion and that determine from the past irradiance measurements the next transmission cycle of sensor values to the cloud. Ref. [14] uses a predictive model for the available energy to guide the operation of the task of a microcontroller. Both approaches are based on predicting solar irradiance based on past data for up to 24 h.

The work from [14] adapted application parameters according to the available energy. Their approach is based on a model predicting the energy over 24 h in intervals of 15 min. The energy management task consumes quite some energy, and the work is limited to continuously powered devices. In [12], the authors applied the Proximal Policy Optimization technique to simulate the energy management features of sensor nodes; in [15], the authors utilized the Reinforcement Learning technique to keep the state of charge of the battery at an optimal level. To avoid too high and too low charging levels, the duty cycle of the application was adapted. The model considers the energy consumption and the currently harvested energy and does not utilize energy predictions.

The authors of [16] developed a control algorithm for medium access in wireless sensor networks. It uses solar and wind energy in the nodes to guarantee operation of the network. The duty cycle is adapted to the currently available renewable energy. The work also does not use any energy prediction.

Our approach applies long-term irradiation prediction, e.g., for two weeks, and can thus ensure extended sensor lifetime by proactively optimizing duty cycles over multiple days before weather changes. The available related works do not use long-term predictions and thus are limited to management in maximum 24 h periods. Notably, our energy neutrality approach is executed on tiny embedded IoT nodes and automatically adapts to the energy consumption characteristics of the application, the microcontroller, the weather prediction, the location of the solar panel, and the changing harvesting efficiency due to time-dependent shadowing and changing irradiance angle—e.g., the seasonal change in the angle in combination with trees might lead to changes in the harvested energy even when the same hourly sunshine is predicted. Our automatic approach significantly simplifies the deployment of solar-based sensors.

## 3. Solar-Based Energy Management in the Serverless IoT Framework

Efficient energy management in IoT-enabled applications enables continuous operation of IoT sensors. This section explains the proposed duty cycle management mechanism using long-term solar irradiation predictions. The proposed energy management approach is integrated in our Serverless IoT Framework.

### 3.1. SIF Framework

*Serverless IoT Framework (SIF)* for IoT applications in the Computing Continuum of sensor devices, edge servers, and cloud data centres leverages serverless computing in the form of *Function-as-a-Service* for programming the device, edge, and cloud layer in a uniform approach [17]. On all the layers, applications are written in an event-based style, where stateless functions subscribe to events and function invocations are scheduled to the best resource for execution.

The management of function invocations enables optimizations in space and time. For example, invocations can be offloaded from sensor devices to the edge in case of energy shortage. On the edge level, invocations can be scheduled onto an FaaS edge cluster, in our case based on Knative, to share resources of multiple geographically near edge servers. Scheduling decisions are based on resource requirements and the maximum response time of the function. The maximum response time allows automatic batching of invocations for resource availability. For example, function invocations for transferring measurements from the sensor to the edge via MQTT require WiFi, which takes a lot of energy for setup and transmission. If all the transfers are required only at the end of the day, multiple transmissions can be combined into a single WiFi session.

The SIF platform orchestrates all the components of SIF applications on the provided resources. It consists of two parts, the *SIF Device Platform (SIF-D)* for IoT devices and the *SIF Edge Cloud Platform (SIF-EC)* for edge servers and the cloud. The conceptual structure of both platforms is the same, while the implementation mechanisms are specialized for the device and the edge-cloud layer.

The TUM SIF testbed consists of passively cooled systems based on i3, i5, i7, and AMD as well as a number of Raspberry Pi 5 and Jetson Orin systems in the edge layer. The devices on the device layer are sensors and actuators based on the ESP32 microcontroller family. The ESP32 combines two Tensilica cores, with an ultra-low power core and WIFI and Bluetooth. It has 520 KB on chip memory and comes with additional flash and PSRAM. Besides ESP32 WROOM 32E and ESP32 WROVER-B with the addition of 8 MB of PSRAM, we also use the ESP32-S3 WROOM-2 with Tensilica 7 CPUs providing vector units, 8 MB PSRAM, and 32 MB of flash.

### 3.2. Energy Management in SIF

**Figure 1:**
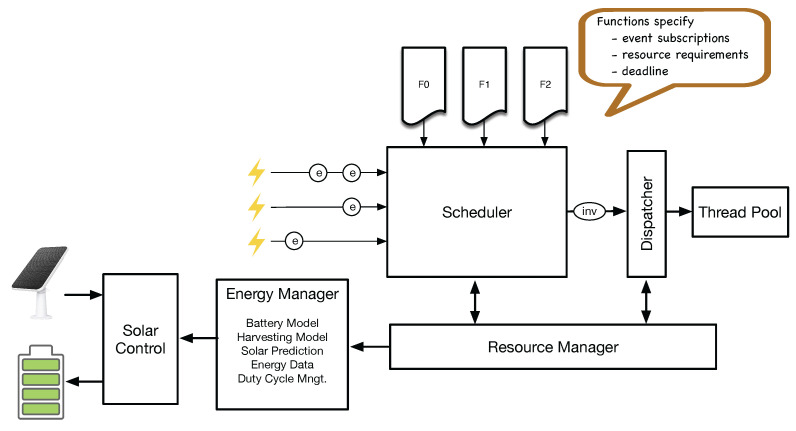
The scheduler of the SIF Device Platform (SIF-D) manages events, event subscriptions, and function invocations of sensor node applications. It forwards function invocations (inv) to the Dispatcher for execution by a thread from the thread pool ones all resources are available. The Resource Manager provides the required information to the scheduler and switches resources to lower energy states when not used. The energy manager keeps track of the battery state based on the Solar Control Board. It also executes the algorithm combining solar prediction and battery state to determine the sleep time that guarantees continuous operation.

This article focuses on the role, functionality, and implementation of the energy manager in SIF-D. SIF-D realizes SIF on microcontrollers used in IoT devices such as ESP32. On the tiny devices, the application components and SIF-D are statically linked as the firmware of the microcontroller. Figure 1 outlines the implementation of SIF for IoT devices. The figure shows the main components implemented in the ESP32 SIF framework. All the components are part of the binary that is flashed to the microcontroller. The application only specifies triggers, events, resources, and functions. The SIF framework provides the runtime system for SIF applications and base classes for the application components.

SIF applications use triggers to generate events that are periodically inserted into the event queue of the scheduler or due to an interrupt. The computation is realized as functions that subscribe to the events and specify the resource requirements and the deadline for invocations. The scheduler creates invocations from the subscription information, checks the availability of resources, and schedules invocations for execution. Once the invocations are scheduled, it is the responsibility of the dispatcher to send the invocations to an executor. On the ESP32, it is a pool of threads that are executed on the two Tensilica cores. The Dispatcher informs the Resource Manager when an invocation is finished and the resources are freed again.

Listing 1 introduces the SIF implementation of the energy manager. The SIF Manager is itself a resource in SIF. An application that uses the energy manager will create the resource and add it to the scheduler and the resource manager.

**Listing 1.** This listing shows the initialization of the Energy Manager.
 
 1 void EnergyManager :: resourceInit
 2 ( config , batteryConfig , harvestingModel , targetRSOC , ...)
 3 {
 4 ...
 5   Max17048 * max17048 = new Max17048 (" MAX17048 ", I2C_0 );
 6   Scheduler :: addResource ( max17048 );
 7   Ina3221 * ina = new Ina3221 (" INA3221 ", I2C_0 , 0 x40 );
 8   scheduler . addResource ( ina );
 9   ...
10   battery = new ( Battery ( batteryConfig ));
11   ...
12   FunctionFactory :: registerFunction
13         ( FunctionType :: CalcDutyFreqF , CalcDutyFreqF :: create );
14   scheduler . subscribe
15         ( EventType :: GetNextDutyFreqE , FunctionType :: CalcDutyFreqF );
16   FunctionFactory :: registerFunction
17         ( FunctionType :: CalcChargingModelF , CalcChargingModelF :: create );
18   scheduler . subscribe
19         ( EventType :: GetChargingModelE , FunctionType :: CalcChargingModelF );
20   ...
21   getNVSlastState ( ... );
22   updateEnergyCounters ( ... );
23
24   if ( time_info . tm_hour == 1
25          || esp_sleep_get_wakeup_cause () ==
                ESP_SLEEP_WAKEUP_UNDEFINED ) {
26   scheduler . enqueue ( new GetNextDutyFreqEvent (( double ) targetRSOC ));
27   }
28   ...
29 }


The energy manager configuration, not shown in the listing, specifies the bounds for the sleep period after a duty cycle and the search steps $SLT=(SLT_{min},SLT_{max},SLT_{step}$ in seconds. It is assumed that $SLT_{min}$ is preferred but a longer sleep time saving more energy can be tolerated up to $SLT_{max}$. $SLT_{step}$ is used in the search for a fitting sleep time. The configuration also specifies the power consumption in deep sleep $P_{sleep}$ in mW because power measurements are impossible while the microcontroller is in deep sleep mode.

Another important configuration is the *charging model*. It specifies the maximum charge power for the given battery and the given solar manager on the solar controller board depending on the Remaining-State-of-Charge (RSOC) of the battery.

The energy manager initialization demonstrates how resources, functions, and events are used in SIF IoT applications. Lines 3–4 add the battery gauge for measuring RSOC to the resource management. The chip is connected to the I2C bus 0. Lines 5–6 add the INA3221 resource for the power measurements of the solar panel, the battery, and the load. Line 8 creates a battery object for the charging model.

Lines 10–13 register two functions with the scheduler and specify the event subscriptions. CalcChargingModelF is triggered via the GetChargingModelE, which is created by the application to trigger a full charging cycle. CalcDutyFreqF computes the sleep time to be used based on the weather prediction. It is discussed in detail in Section 4.4.

Line 15 retrieves the last state of the energy manager from the flash memory of the microcontroller, for example, the sleep time ($T_{sleep}$ in seconds) and the last panel power $P_{panel}$. Line 16 updates the energy counters (mWh) of the three channels:

- $E_{load}=\left(E_{load}+P_{sleep}\times T_{sleep}\right)/3600$.
- $E_{panel}=\left(E_{panel}+P_{panel}\times T_{sleep}\right)/3600$.
- $E_{bat}=\left(E_{bat}+P_{bat}\times T_{sleep}\right)/3600$.

Finally, lines 17–19 trigger the recalculation of $T_{sleep}$ based on the solar prediction and $RSOC_{target}$. The target RSOC is the battery state-of-charge at the end of the prediction period—e.g., after 5 days the battery should still have 20% of its capacity. The event triggering the recalculation is created if the current time is 1 a.m. or the reboot was not triggered by a wakeup from deep sleep.

### 3.3. Solar Control Board

**Figure 2:**
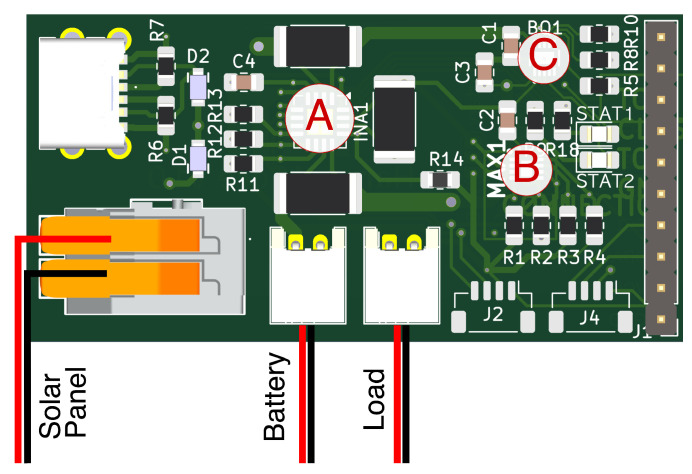
TUM developed this board from off-the-shelf components. It combines a three channel power IC (INA3221) (A), a battery gauge IC (MAX17048) (B), and a solar management IC (BQ25185) (C).

**Figure 3:**
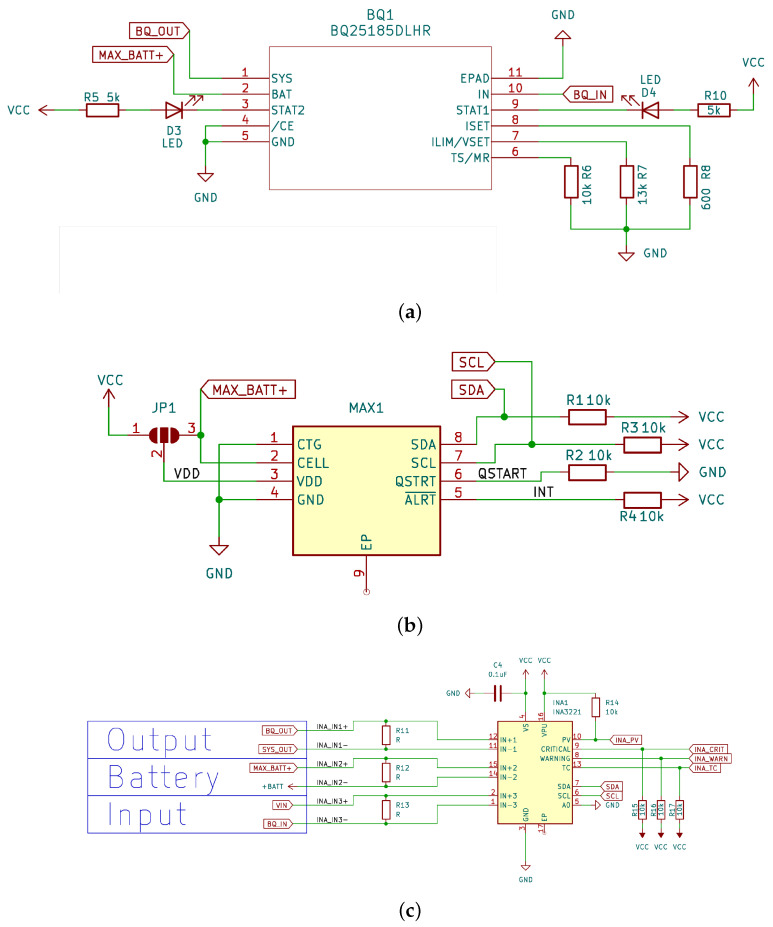
Schematics of the solar control board. (**a**) Schematics for the solar management chip (BQ25185). BQ24074 was replaced by BQ25185 due to chip availability. (**b**) Schematics for the battery gauge (MAX17048). (**c**) Schematics for the power measurement chip (INA3221).

The energy neutrality system integrates a solar panel as the primary energy source, a rechargeable lithium-ion battery for energy storage, and a custom-designed printed circuit board (PCB) (see Figure 2 and Figure 3) featuring three main components:

(a) A three-channel power monitoring IC (INA3221).
(b) A battery gauge (MAX17048).
(c) A solar charger IC (BQ24074).

#### 3.3.1. Three-Channel Power Monitor (INA3221)

The INA3221 IC measures voltage and current simultaneously on three distinct channels: the solar panel input, battery input and output, and system load. This comprehensive monitoring capability enables accurate real-time tracking of power usage and generation. Data from the INA3221, transmitted via an I^2^C interface, are used in the optimization of the duty cycle frequency.

The INA3221 resource created by the energy manager creates a separate thread that measures every 20 ms the power consumption of each channel and accumulates the energy counters for $E_{panel}$, $E_{bat}$, and $E_{load}$. When the resource terminates, the current energy values are stored in the FLASH and reloaded when the resource is created again. Thus, the measurements are not lost during deep sleep. As presented in Section 3 the counters are corrected according to the time in deep sleep when the energy manager is initialized.

#### 3.3.2. Battery Gauge (MAX17048)

Accurately determining the battery’s state-of-charge (SoC) is crucial for effective energy management, particularly for the calculation of the energy-aware duty cycle management. The MAX17048 battery gauge utilizes Maxim’s ModelGauge™ algorithm, which estimates the battery’s SoC by continuously monitoring battery voltage dynamics, including open-circuit voltage and rate of voltage change; the MAX17048 provides precise SoC estimations. Its algorithm can also adapt to battery ageing and usage patterns, ensuring sustained accuracy throughout the battery’s operational life. Such adaptability is crucial for predicting battery performance and energy availability, directly influencing power allocation and scheduling decisions within the IoT system.

#### 3.3.3. Solar Charger (BQ24074)

The Solar Charger manages energy distribution from solar panels and the battery to the load. It addresses challenges associated with photovoltaic systems, such as fluctuating output voltages and power path management to constantly power the load, even during low-sunshine periods.

Solar Panels follow an I-V-Curve where the output varies given different lighting conditions and a current. They also have the issue of a voltage collapse if the drawn current becomes too large. Instead of Maximum Power Point Tracking (MPPT), the BQ24074 IC employs Input Dynamic Power Management (VIN-DPM). It follows the USB standard of keeping the input voltage at 5 V and around 0.5 A. MPPT is more efficient at higher voltages, but if the solar panel voltage is only 1 V above the battery voltage, the added complexity of DC/DC converters increases cost and is not more efficient than linear converters at lower voltages. The Input Dynamic Power Management has a voltage-based loop that regulates the current so the voltage does not drop below 4.5 V, preventing a voltage collapse [18]. Additionally, Dynamic Power Path Management (DPPM) provides precise control and adaptive power allocation between the battery, load, and solar source.

The solar control board uses the Solar Charger (BQ24074) for its own low-power requirements. This is important if the ESP32 is in deep sleep consuming only 0.032 mW. While the BQ24074 only consumes 0.051 mW, the two other boards shown in Table 1 consume 2.781 mW and 8.248 mW, respectively. This efficiency of the Adafruit BQ24074 [18] is primarily due to the lack of voltage conversion, as both the input and output voltages are identical at 4.14 volts. Both other boards, the DFRobot (CN3165) [19] and Waveshare (CN3791) [20], apply voltage conversions, which leads to a much higher quiescence current.

To optimize energy harvesting hardware, we chose the BQ24074 IC for a very low energy consumption and loss due to similar voltages and no conversion, as well as a comparable performance of solar output maximization to MPPT.

The total material cost for manufacturing one control board was USD 14.19 per unit for a small series of five boards and the PCB production cost for five boards including the stamp was USD 17.11.

## 4. Energy-Aware Duty Cycle Management

To ensure continuous operation for solar panel-based IoT sensors under varying weather conditions, the energy manager in SIF-D implements an energy-aware duty cycle management. It uses weather prediction information to adapt the duty cycle frequency so that after a given number of days the remaining state of charge of the battery is above a certain threshold.

The management algorithm is based on the following parameters:

ND: Forecasting days—This parameter specifies the number of days to be covered in the computation. The first day is always today, the second is tomorrow, and so forth.
$SLT=(SLT_{min},SLT_{max},SLT_{step})$: Sleep time interval in seconds. Preferred is the highest duty cycle frequency, i.e., the minimum sleep time between duty cycles. $SLT_{max}$ defines the lowest tolerable frequency, and $SLT_{step}$ the granularity of the search for the best duty cycle frequency that guarantees the RSOC threshold.
$RSOC_{init}$: Initial Battery Level—To predict the energy availability over the forecasting period, the current state of charge of the battery is considered.
$RSOC_{target}$: Target Battery Level—This parameter defines the minimum battery level of the device at the end of the forecasting period.

It utilizes several models in the energy-aware duty cycle management algorithm. These models are introduced in the following subsections.

### 4.1. Solar Energy Harvesting Model

The proposed system uses an *energy harvesting model* that determines the energy harvested from the attached solar panel in the forecasting period with hourly granularity.

(1) $$E_{harv}\left(d,h\right)=E_{irrad}\left(d,h\right)\times Efficiency_{panel}\left(h\right)$$

This combines two models: (a) the *solar irradiance prediction model* predicting solar irradiance from a weather forecast service for the forecasting period, and (b) the *panel efficiency model* capturing the characteristics of the panel, its placement, and the seasonal efficiency due to different solar angles.

*Solar irradiance* is a measure that describes the density of solar radiation falling on a surface, with the unit being W/m^2^. It is an important metric, giving us an upper bound on what is theoretically possible to harvest.

According to the solar irradiance forecasting service Solargis [21], Munich, Germany, has an annual irradiation of 1144.3 kWh/m^2^, meaning that the sun generates 1144.3 kWh of energy per square meter.

Irradiance predictions can be obtained from the Open WeatherMap (openweather.co.uk, accessed on 15 July 2025) service’s Cloudy Sky model. It combines the raw irradiation with the predicted cloud coverage for the target region. In our test we used a different approach since the service focuses on large solar panels and the precision in kWh/m^2^ is too low for small-sized panels used in IoT devices.

Our approach for predicting solar irradiance is based on the information from the German Weather Service (dwd.de, accessed on 15 July 2025). It predicts the sunshine duration in minutes per hour $T_{sun}\left(d,h\right)$. We combine this solar irradiance prediction model with the panel efficiency model into the energy harvesting model.

The *panel efficiency model* captures the panel efficiency, its placement, and seasonal variation. This covers, for example, the angle of the panel, shadow periods due to surrounding trees or other obstacles, as well as the path of the Sun at a certain time of the year. To simplify the deployment and calibration of the panel, we capture the harvested energy on a bright day (predicted continuous sunshine from sunrise to sunset) with an hourly resolution $E_{max}$ (hour). This model has to be updated periodically (e.g., per month) to adapt to seasonal variations.

Thus the harvesting model used in our testbed is

(2) $$E_{harv}\left(d,h\right)=\left(E_{max}\left(h\right)/60\right)\times T_{sun}\left(d,h\right)$$

where d∈[1…ND] and h∈[0…23]. $E_{max}\left(h\right)/60$ is the maximum harvested energy per minute and is multiplied with sunshine duration in minutes.

The predicted sunshine duration is given in minutes and multiplied by the maximum energy per minute for bright sunshine.

### 4.2. Energy Consumption Model

The operation of IoT devices is modeled as a periodic wakeup of the device to perform sensor measurements and other tasks. The time the device is active is called the duty cycle $T_{duty}$ and the rest of the time is the sleep time $T_{sleep}$. While the power consumption $P_{duty}$ can be measured with the attached board, the power consumption in deep sleep $P_{sleep}$ has to be given as a parameter since in deep sleep the microcontroller is not able to measure energy via the solar control board.

Assuming that $T_{duty}+T_{sleep}<<$ 3600 s, the hourly used energy can be calculated as mentioned in Equation (3):

(3) $$\begin{array}{cc} E_{load}^{T_{sleep}}= & (P_{duty}\times T_{duty}+P_{sleep}\times T_{sleep})\times \\ & \left(3600/\left(T_{duty}+T_{sleep}\right)\right) \end{array}$$

### 4.3. Battery Charging Model

The harvested energy is used to cover the consumed energy as well as surplus energy to charge the battery. Depending on the RSOC value of the battery, charging has a different efficiency. The battery charging model determines the maximum charging power $P_{charge}\left(RSOC\right)$ depending on the battery’s RSOC. The higher the RSOC, the less power can be charged to the battery.

To capture the charging model of the used battery, a full charging cycle is executed, and periodic measurements of RSOC and the charging power are performed via the function CalcChargingModelF while the battery is charged by the solar manager on the solar control board via a power supply.

The energy that can be maximally charged per hour for a given RSOC and a given surplus power is limited by the following formula:

(4) $$E_{charged}=min\left(P_{charge}\left(RSOC\right),P_{surplus}\right)\times 1h$$

### 4.4. Duty Cycle Optimization

Algorithm 1 specifies the strategy used to select the sleep time $T_{sleep}^{d}$ between two duty cycles for each day *d* so that at the end of the forecasting period of ND days, the $RC_{target}$ is guaranteed.
**Algorithm 1:** Compute Duty Frequency
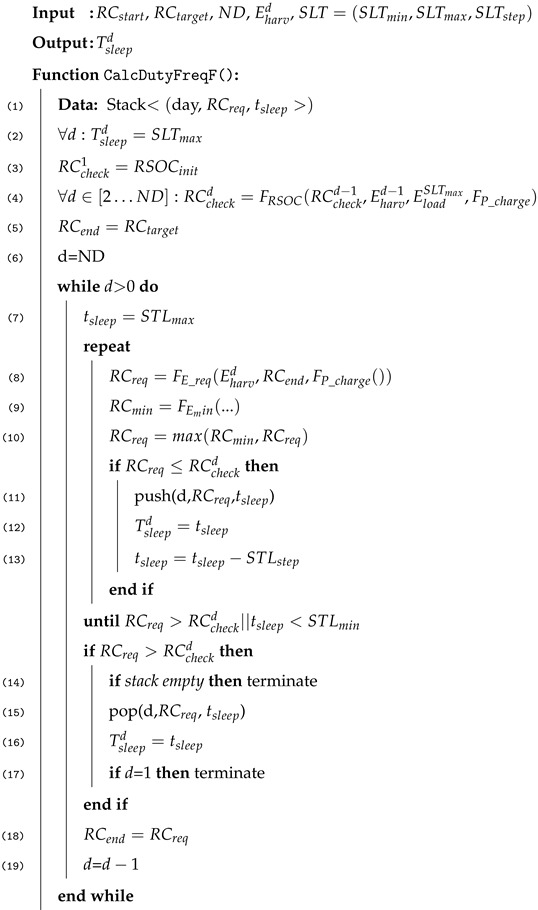


It uses a stack of valid sleep times per day. A sleep time is called *valid* for a day *d* if $RC_{target}$ can be met under the assumption that RSOC at the beginning of day *d* is larger than a given required RSOC $RC_{req}$ for that day. The algorithm uses backtracking through valid configurations to find a valid setting $T_{sleep}^{d}$ for all days.

Line 2 initializes the sleep time for all days to $SLT_{max}$.

Lines 3–4 initialize the checkpoints at the beginning of each day. The checkpoint captures the best RSOC at the beginning of each day based on the predicted harvested energy and the energy consumed by the load on that day for $SLT_{max}$. $RC_{check}^{1}$ for the first day is the RSOC measured by our solar control board when the algorithm is executed. For each following day, RSOC is calculated by $F_{RSOC}\left(RC_{check}^{d-1},E_{harv}^{d-1},E_{load}^{SLT_{max}},F_{P\_charge}\right)$. It determines the maximum possible RSOC on the next day based on the RSOC of the previous day, the predicted harvested energy, the energy draw of the load for the longest sleep time, and the charging model.

Line 5 initializes $RC_{end}$ to $RC_{target}$. This is the value to be guaranteed at the end of a day in the following while loop.

The while loop computes $T_{sleep}^{d}$ starting from the last day and going back to Day 1. It terminates either on line 14 if no valid sleep time for a day was found and no possible low-energy configurations for later days are on the stack. In that case, the maximum sleep time is used to save as much energy as possible. There is still hope that the weather improves and more energy can be harvested as is currently predicted. If a valid sleep time combination $T_{sleep}^{d}$ was found, the algorithm terminates on line 17.

For each day, the algorithm starts with $STL_{max}$ and searches in the repeat loop for the shortest sleep time guaranteeing $RC_{end}$ and not exceeding the RSOC checkpoint. All found valid settings are pushed to the stack (line 11) so that, if an earlier day has no valid solution, energy can be saved on this day by popping a longer sleep time. Function (line 8) $F_{E\_req}\left(E_{harv}^{d},RC_{end},F_{P\_charge}\left(\right)\right)$ computes the required energy at the start of the day. It simulates the energy flow with an hourly granularity backwards from 11 p.m. to 0 a.m. Function $F_{E\_min}$ computes the minimal required energy at the start of the day. It can happen that this is larger than taking the harvested energy into account, since in the first night hours no harvesting is possible.

The repeat loop terminates either with the shortest sleep time or with the sleep time that first requires more energy at the start of the day, as guaranteed by the checkpoint. If it terminates after finding a solution for the shortest sleep time, the algorithm sets $RC_{end}$ on line 17 and continues with the previous day. In the latter case, it checks whether the stack is empty. If not, the last valid setting is popped from the stack. It can be the current day or any previous day. If this is a valid solution for the first day, a valid combination $T_{sleep}^{d}$ is found. Otherwise, the algorithm continues from the day before the day with the popped valid solution.

The stack of valid sleep times significantly speeds up the algorithm, as the calculation of $RC_{req}$ and $RC_{min}$ need not be repeated.

#### Algorithm—An Exploratory Study

The proposed algorithm is explained with an example as follows: Assume that the forecasting period is three days (ND=3) into the future, namely, today (Day 1), tomorrow (Day 2), and the day after tomorrow (Day 3).

At the beginning of the first day, the device has a battery level of 60% ($RC_{start}$) and wants to finish executing tasks with at least 30% of battery charge ($RC_{target}$) the day after next (i.e., end of day three). Algorithm 1 starts by setting the battery checkpoints between each day to 50% at the beginning of Day 2 and then to 40% at the beginning of Day 3.

Additionally, we have defined three duty cycle frequencies, namely, 65, 45, and 25 s, i.e., SLT=(25,65,20). The algorithm starts searching for the shortest sleep time at the end of the last day (Day 3). To do so, it chooses the longest sleep time and calculates how much battery level is needed to reach the specified goal. It reduces the sleep time until the needed battery level surpasses the checkpoint.

In the example, the 40% battery level checkpoint is not surpassed by the 65-second duty cycle frequency. This valid sleep time is pushed to the stack as D3/35/65 (which represents Day 3, a 65 s duty cycle frequency, and a battery level of 35% at the beginning of Day 3). The checkpoint is surpassed with a 45 s duty cycle frequency.

After reaching the checkpoint, the algorithm continues with Day 2 (tomorrow). On Day 2, the algorithm starts again with the longest sleep time and pushes (D2/40/65) to the stack. Since also the sleep time of 45 s is a valid setting with a required RSOC of 48%, but 25 s surpasses the checkpoint of 50%, the calculation for Day 1 starts with $RC_{end}=48$. Thus, the 45 s of sleep time is used for Day 2, and the algorithm continues with Day 1. On Day 1, after calculating with the longest sleep time, the required battery level is 66%. This would mean that at least 66% is required to reach the target battery level of 30%, but only 60% is available.

The algorithm needs to backtrack and pops the last state of Day 2 with a frequency of 65 s and the required battery level of 40% (D2/40/65). Now, Day 1 is recomputed, and the longest sleep time of 65 s requires only 55%, and a valid solution is found. A sleep time of 45 s will also be tried but will again exceed $RC_{start}$ and is thus not valid.

Accordingly, the algorithm terminates with the following calculated duty frequencies: $T_{sleep}^{1}=65\;s$; $T_{sleep}^{2}=45\;s$; and $T_{sleep}^{3}=45\;s$.

## 5. Experimental Results

To validate the techniques presented in the paper for automatically adapting the duty cycle frequency to the predicted solar energy generation, we created a test bed consisting of an ESP32 development board [22] that is connected to our solar control board. We used a 5000 mWh LIPO battery and a 112 square millimeter sized solar panel.

The application used in all our experiments collects periodic temperature values and communicates energy and power information for the panel, the load, and the battery together with the measured temperature to our cloud data platform.

While during the duty cycle the energy values were measured via the solar control board, the energy was estimated during deep sleep as presented in Section 3. The sleep time is precisely measured based on the continuously running real-time clock.

### 5.1. Battery Charging Model

**Figure 4:**
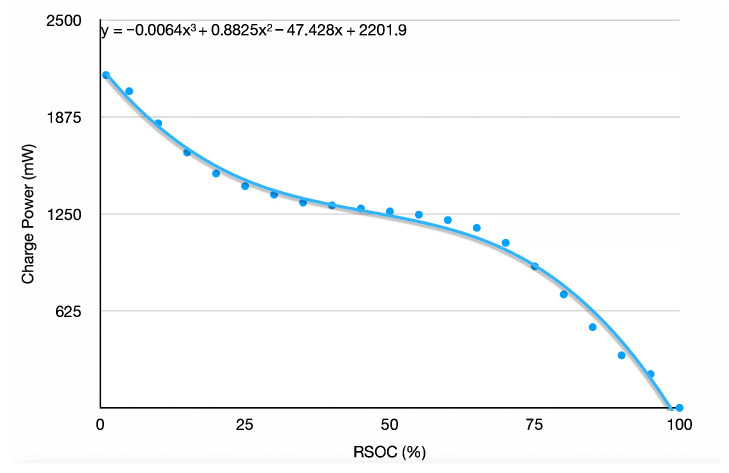
The charging model of the 5000 mWh LIPO battery. It presents the cubic function determined by curve fitting and used as the $F_{pcharge}$ in the experiments.

The test device uses an LIPO battery with a capacity of 5000 mWh. We created the battery charging model by continuously charging the battery from a power supply connected to the solar control board and measuring RSOC and power every 10 ms. The raw measurements were then turned into the prediction function by curve fitting. The result is shown in Figure 4.

### 5.2. Importance of Duty Cycle Frequency Management

**Figure 5:**
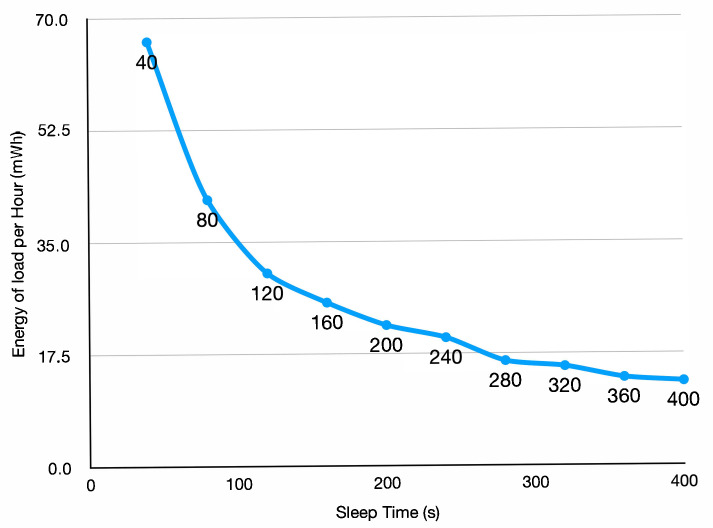
Energy per hour of the load depending on the sleep time.

Figure 5 presents the energy consumption per hour of the load when varying the sleep time between 40 and 400 s. For these measurements an external multimeter (Otii ARC (www.qoitech.com/otii-arc-pro accessed on 15 July 2025) was used to collect power values from the battery. The solar panel was removed for those tests.

### 5.3. Panel Efficiency Model

The energy-aware duty frequency management is based on predicting the harvested energy from a solar panel from the predicted sunshine duration, in our case the DWD. This sunshine duration is turned into the harvested energy based on the panel efficiency model.

**Figure 6:**
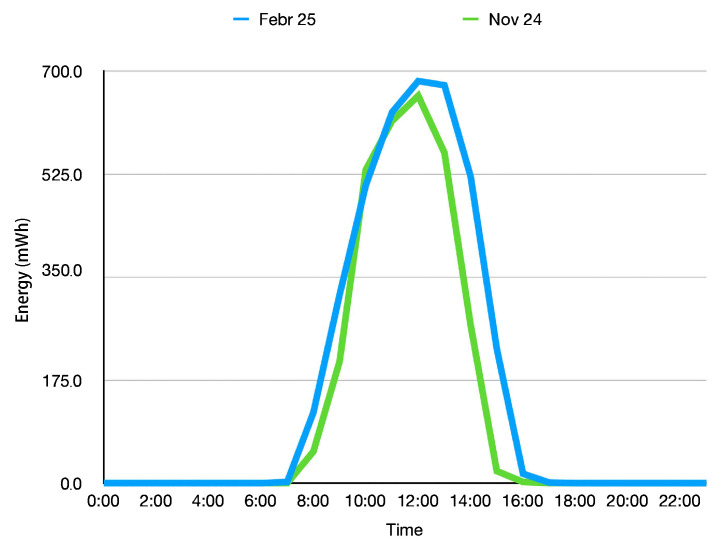
The energy harvested from the solar panel used in our experiments changes over the year. This diagram shows the models obtained in November 2024 and February 2025.

Figure 6 shows the energy harvested in one hour from the solar panel on a day with full sunshine. This model is then used to compute the harvested energy based on the predicted minutes of sunshine per hour from the weather service. We can observe that the curve in February shows higher values and also an extended period of energy harvesting.

### 5.4. Solar Energy Harvesting Model

**Figure 7:**
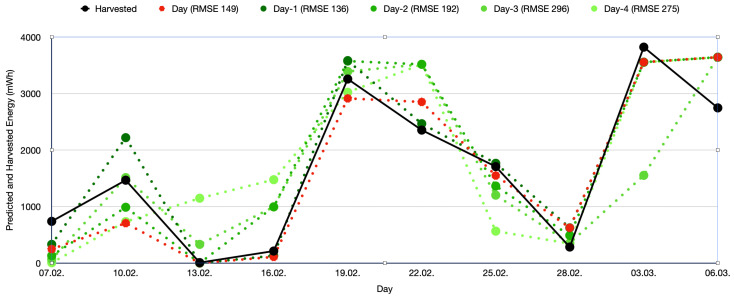
Prediction quality for each day. The graph shows the harvested energy in black, the predicted energy for the given solar panel and position on that day in red, and the prediction on the days before in greenish color.

Figure 7 shows the prediction quality for 7 February to 6 March 2025. The black curve is the finally harvested energy on that day. The red curve is the energy predicted for that day on that day. The four green curves show the energy predicted for the day one to four days ahead. The deviations show the quality of the sunshine prediction from the weather service. The sunshine minutes take into account the cloudiness, but there are many other factors, such as the humidity, that influence the irradiation. It is interesting to see that the predictions are quite accurate. The legend also shows the RMSE for all predictions with a certain number of days ahead. The prediction quality improves the fewer days ahead the sunshine duration is predicted.

### 5.5. Duty Cycle Adaptation

**Figure 8:**
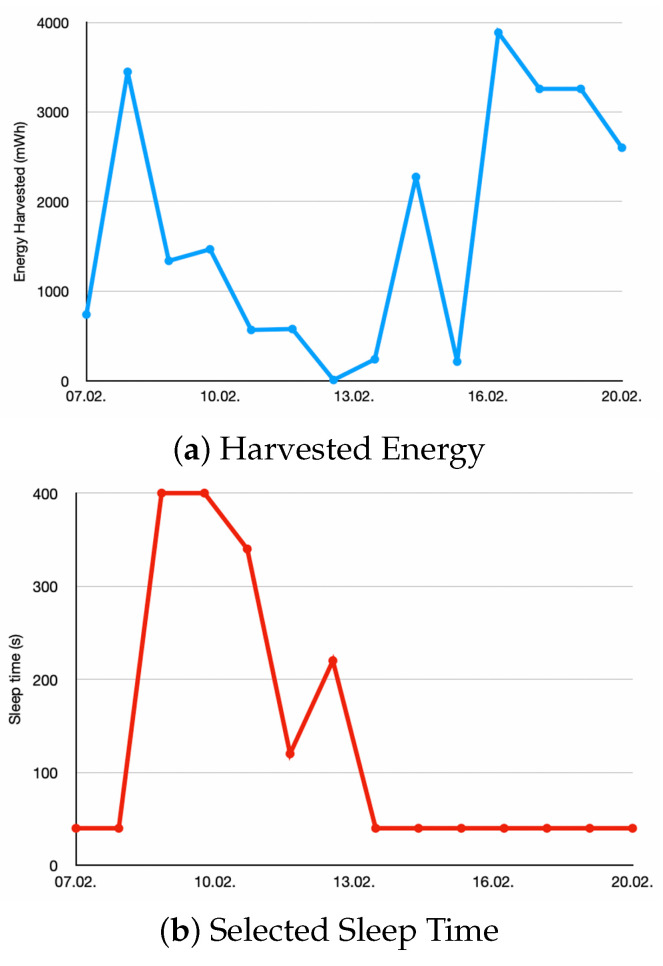

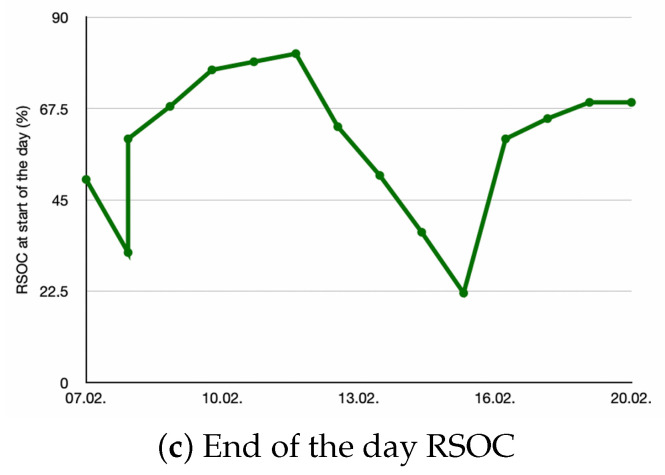
These graphs present (**a**) the harvested energy per day, (**b**) the selected sleep time, and (**c**) the resulting RSOC at the beginning of each day for 7–20 February 2025.

Figure 8 demonstrates the application of energy-aware duty frequency management. The curves show the chosen sleep time together with the harvested energy and the battery RSOC.

The settings for the algorithm are STL=(40,400,20) and $RSOC_{target}=20\%$. The blue curve shows the harvested energy, which is decreasing over the first days and then increasing again. The red curve shows the chosen sleep time. The algorithm switched from a sleep time of 40 s to 400 s based on the predicted decrease of the harvested energy. It then gradually decreases the sleep time as the prediction improves. The green curve shows the RSOC at the beginning of each day. It is measured at 01:00 when the algorithm setting the sleep time for that day is executed. The curve shows that although the harvested energy reduces, an RSOC of over 20% is achieved.

If our predictive duty cycle management had not been used, the sleep time would not have been increased proactively and the IoT device would have run out of battery after 10 February.

### 5.6. Integration of the Sleep Time Settings

The application used in these tests is programmed in SIF and instantiates the energy manager. The entire working of the energy manager is transparent to the application. It only provides the STL settings and the power consumption in deep sleep to the manager. Following the event-based programming in SIF, the application registers a function that subscribes to SIF_AdustDutyCycleFreqE event. This event carries the computed sleep time setting. The application function then adapts the trigger for the temperature measurement to fire only according to the new sleep time.

The whole approach is based on the automatic deep sleep implemented in SIF-D [17]. Whenever no events are available and no function invocations are waiting for execution or are currently executed, the SIF scheduler computes the time until the next trigger will fire and switches the microcontroller to deep sleep for an appropriate time. The microcontroller is then woken up early enough to start all the used resources and then handle the upcoming trigger.

This approach in SIF allows easily adapting the sleep time according to the computed value of the energy manager in SIF applications.

## 6. Conclusions

This paper presented a novel approach to guarantee continuous operation of IoT devices. It is based on energy harvesting through solar panels and extends the traditional approach of estimating the number of low-energy days and selecting the battery accordingly. Our approach allows us to reduce the battery size by automatically adapting the duty cycle frequency to the predicted harvested energy. It chooses the sleep time to guarantee a predefined required battery RSOC target after a forecasting period of a certain number of days.

The implementation is carried out in the Serverless IoT Framework (SIF) and specifically on the SIF-D platform for sensor devices. It outlines the models that are required for the energy-aware duty cycle frequency management and the algorithm used to compute the sleep time for the actual day and the days in the forecast period. The integration of the adaptation of the sleep time in SIF applications is based on the general programming mode with event-triggered functions.

The algorithm uses the measurements and the solar management provided by a specialized solar control board developed at TUM based on off-the-shelf, low-cost components. It combines energy measurements during the duty cycle through the SIF energy manager with estimated values based on the measurements during the last duty cycle and the sleep time to update the energy counters accordingly at the beginning of the next duty cycle.

## Figures and Tables

**Table 1:** Quiescent (self-consumed) power of three commercial solar-charger breakout boards when the ESP32 is in deep sleep.

Charger	Conversion	Output	Input	Load	Extra Power
	Type	Voltage	Power	Power	Consumed
			(mW)	(mW)	(mW)
Adafruit (BQ24074)	No	4.14 V	0.083 mW	0.032 mW	0.051 mW
DFRobot (CN3165)	Boost (up)	5 V	2.82 mW	0.034 mW	2.781 mW
Waveshare (CN3791)	Buck (down)	3.3 V	8.28 mW	0.032 mW	8.248 mW

## Data Availability

The original contributions presented in this study are included in the article. Further inquiries can be directed to the corresponding author.
